# In a pursuit of optimal glycan fluorescent label for negative MS mode for high-throughput N-glycan analysis

**DOI:** 10.3389/fchem.2022.999770

**Published:** 2022-10-03

**Authors:** Dinko Šoić, Zvonimir Mlinarić, Gordan Lauc, Olga Gornik, Mislav Novokmet, Toma Keser

**Affiliations:** ^1^ Faculty of Pharmacy and Biochemistry, University of Zagreb, Zagreb, Croatia; ^2^ Genos Glycoscience Research Laboratory, Zagreb, Croatia

**Keywords:** N-glycans, label, derivatization, fluorescence, mass spectrometry, negative mode, high-throughput

## Abstract

Over the past few decades, essential role of glycosylation in protein functioning has become widely recognized, rapidly advancing glycan analysis techniques. Because free glycan’s lack chromophore or fluorophore properties, and do not ionize well, they are often derivatized to facilitate their separation or detection, and to enhance the sensitivity of the analysis. Released glycan’s are usually derivatized using a fluorescent tag, which enables their optical detection in LC profiling. Some fluorescent labels can also promote ionization efficiency, thus facilitating MS detection. For this reason, there is a need to design fluorophores that will contribute more to the fluorescence and ionization of glycan’s and the need to quantify these contributions to improve glycan analysis methods. In this paper we focused on negative MS mode as these methods are more informative than methods involving positive MS mode, allowing for a less ambiguous elucidation of detailed glycan structures. Additionally, traditional glycan labels in negative mode MS usually result with diminished sensitivity compared to positive mode, thus making selection of appropriate label even more important for successful high-throughput analysis. Therefore, eleven fluorescent labels of different chemo-physical properties were chosen to study the influence of label hydrophobicity and presence of a negative charge on glycan ionization in negative MS mode. N-glycans released from IgG sample were labeled with one of the eleven labels, purified with HILIC-SPE and analyzed with HILIC-UPLC-FLR-MS. To make evaluation of studied labels performance more objective, analysis was performed in two laboratories and at two mobile phase pH (4.4 and 7.4). Although there was a notable trend of more hydrophobic labels having bigger signal intensities in one laboratory, we observed no such trend in the other laboratory. The results show that MS parameters and intrinsic configuration of the spectrometer have even bigger effect on the final ESI response of the labeled-glycan ionization in negative MS mode that the labels themselves. With this in mind, further research and development of fluorophores that will be suitable for high-throughput glycan analysis in the negative MS mode are proposed.

## Introduction

Glycans, as a type of carbohydrates are, along with proteins, nucleic acids and lipids, one of the four main building blocks of the living world. Besides serving constructive, storing, or protective roles, with evolution, glycans became increasingly more complex and began to acquire other functional roles crucial for complex organisms (Lauc et al., 2014). Improper glycosylation underlies many diseases (Freeze, 2006) and some glycans may be used as novel predictive or diagnostic biomarkers for some of them (Cvetko et al., 2021). Therefore, it is important to include high-throughput glycan analysis in different studies to have a complete picture of what is happening. Furthermore, glycans have also been drawn into the spotlight in the biopharma industry, where they are denoted as a critical quality attribute for therapeutic proteins that are glycosylated, such as monoclonal antibodies or erythropoietin (Delobel, 2021).

Glycan analysis usually includes enzymatic or chemical cleavage from the corresponding glycoprotein followed by its separation and purification from the matrix which simplifies the subsequent analysis (Varki et al., 2015). However, free glycans do not have a chromophore which results in poor signal intensities in most analytical techniques. Furthermore, glycans are highly hydrophilic molecules which in most cases impair their ionization in mass spectrometry (MS). Therefore, glycans are often derivatized, the most common being labeling through the reaction of reductive amination where the amino group of a label reacts with an aldehyde group on the reducing terminus of a free glycan followed by the *in situ* reduction of a resulting imine to yield a more stable secondary amine (Anumula, 2006; Ruhaak et al., 2010). The introduction of a glycan label can increase both signal intensity and enhance ionization in MS thus enabling more sensitive glycan analysis (Zhou et al., 2017; Wang et al., 2018). Commonly used glycan labels are 2-aminobenzoic acid, 2-aminobenzamide, 2-aminopyridine, procainamide, and RapiFluor-MS although many others have been developed (Ruhaak et al., 2010; Keser et al., 2018). Furthermore, the glycan label reacts with the free glycan exclusively in a 1:1 ratio which enables easy quantification by measuring the fluorescence.

Mass spectrometry has recently become a preferred method of choice in high-throughput glycomics as it provides accurate glycan identification and quantitation (B. Lebrilla, 2013; de Haan et al., 2020). Structural characterization of N-glycans in MS relies on their fragmentation pattern, which happens by two predominant processes: glycosidic cleavage between the monosaccharide residues, and cross-ring cleavage within the sugar ring. Glycosidic cleavages yield information about the glycan sequence, whereas the cross-ring cleavages provide substantial information on linkage and branching and are crucial for complete elucidation of glycan structure (Harvey, 2020; Grabarics et al., 2022). It has been shown that the negative MS mode offers more structural information on glycan structure than the positive MS mode. This happens because in the negative mode there are relatively more fragments produced by a cross-ring cleavage than the fragments from the glycosidic cleavage, which are relatively more abundant in the positive MS mode. Thus, positive MS mode frequently requires additional step of glycan permethylation and assignment of free hydroxyl groups that are generated by fragmentation in order to obtain linkage information and glycan structure (Harvey, 2020). On the other hand, exact glycan structure in negative MS mode can be obtained using structure-specific diagnostic ions which are present only in negative mode MS/MS spectra due to many cross-ring cleavages (Morimoto et al., 2015). These diagnostic fragment ions of high specificity can distinguish features such as arm position and sialyl linkage, facilitating glycan isomer discrimination (Ashwood et al., 2018). Nevertheless, analysis is hampered by the fact that neutral glycans are less ionized in this mode compared to the positive mode (Ni et al., 2013). Furthermore, most labels in high-throughput glycomic methods are used and optimized for positive MS mode, with an exception of 2-aminobenzoic acid, which is the most commonly and traditionally used label for negative MS mode (Pabst et al., 2009; Trbojević-Akmačić et al., 2022). Despite being less widely performed, recent advances guarantee the place of negative mode MS in glycan analysis (Hykollari et al., 2021). Therefore, there is a need to design fluorophores that will contribute more to the ionization of glycans and to quantify these contributions to improve glycan analysis methods in the negative mode.

It may seem obvious that the good glycan label for the negative MS mode should be the molecule that holds motifs that can readily be deprotonated. However, these acidic motifs usually include electronegative atoms which greatly reduce the lipophilicity of the molecule, thus reducing the ionization. This happens because, with electrospray ionization, more lipophilic molecules are more oriented towards the surface of the droplet (Shuford and Muddiman, 2011). Thus, it is more probable that, during the solvent desorption, these molecules will be contained in increasingly smaller droplets and finally be ionized and sent to the detector (Fenn, 1993). This effect has been observed with many different molecules (Cech and Enke, 2000; Null et al., 2003).

It can be said that there is an interplay between the effects of acidic groups which are readily deprotonated and lipophilicity which increases ionization (Walker et al., 2010). Thus, there is a need to quantify these effects to determine the optimal lipophilicity and negative charge of a potential glycan label for negative MS mode. Therefore, the aim of this study is to measure the ionization of different glycan labels to see which structural characteristics of a molecule affect glycan ionization the most.

## Materials and methods

### IgG sample

IgG sample used for all experiments was isolated and pooled from human plasma of healthy individuals by affinity chromatography using protein G monoliths according to a previously published protocol (14). Briefly, 70 μl of plasma was diluted 10× with PBS, applied to the protein G plate (BIA Separations, Ljubljana, Slovenia) and instantly washed. IgGs were eluted with 1 ml of 0.1 M formic acid and neutralized with 1 M ammonium bicarbonate and then pooled afterwards. A NanoDrop (Thermo Fisher Scientific, Waltham, MA, United States) spectrophotometer was used to measure IgG concentration of the IgG pool. The plasma samples were taken from healthy volunteers and the study was approved by the Ethical Committee of the University of Zagreb Faculty of Pharmacy and Biochemistry, Zagreb, Croatia. All subjects gave written informed consent in accordance with the Declaration of Helsinki.

### Glycan labeling with different labels

Eleven different fluorescent glycan labels were tested, all in pentaplicates: 2-aminobenzamide (2AB), 2-aminobenzoic acid (2AA), 3-aminobenzoic acid (3AA), 4-aminobenzoic acid (4AA), aniline (AN), 4-aminophenol (4AP), benzocaine (BC), 1-naphthylamine (1NA), 3-amino-2-naphthoic acid (32ANA), 6-amino-2-naphthoic acid (62ANA) and 8-amino-2-naphthalenesulfonic acid (82 ANSA) (Table 1). All labels were obtained from Sigma-Aldrich, St. Louis, MO, United States. LogP values were calculated using SwissADME online platform (http://www.swissadme.ch/) (Daina et al., 2017)—consensus logP calculated as an arithmetic mean of logP values generated using five different methods was used. pKa values were calculated using Chemicalize online platform (https://chemicalize.com/) (Chemaxon, Budapest, Hungary). Percentage of ionization was calculated based on the pKa values using the Henderson-Hasselbalch equation: $pH={pK}_{a}+\frac{\log\left[A^{-}\right]}{\log\left[HA\right]}$ .

**TABLE 1:** Glycan fluorescent labels used in the experiment and their chemo-physical properties. LogP values were calculated using SwissADME online platform, while the pKa values were calculated using Chemicalize online platform (Chemaxon).

Name	Abbreviation	Structure	FLR excitation and emission wavelengths/nm	LogP	pKa
2-aminobenzamide	2AB	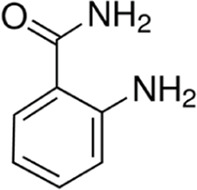	250; 428	0.51	NA
2-aminobenzoic acid	2AA	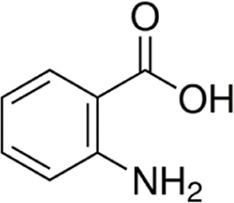	365; 434	0.72	4.89
3-aminobenzoic acid	3AA	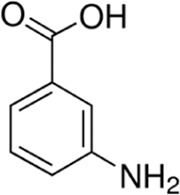	310; 405	0.58	4.81
4-aminobenzoic acid	4AA	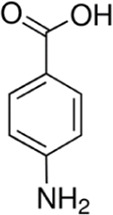	310; 365	0.6	4.77
aniline	AN	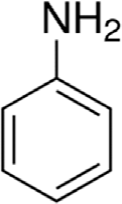	245; 335	1.22	NA
4-aminophenol	4AP	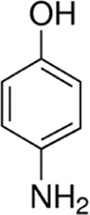	310; 365	0.68	10.4
benzocaine	BC	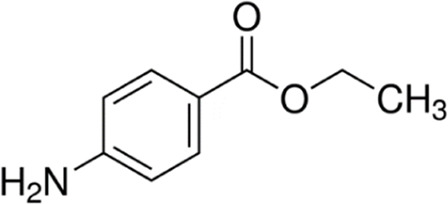	310; 365	1.63	NA
1-naphthylamine	1NA	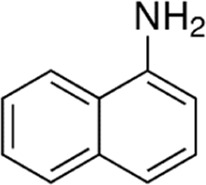	310; 434	2.23	NA
3-amino-2-naphthoic acid	32ANA	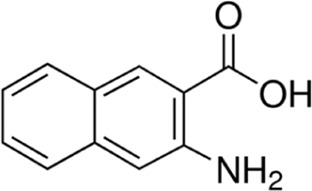	272; 495	1.72	4.76
6-amino-2-naphthoic acid	62ANA	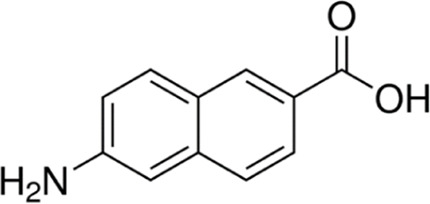	365; 434	1.92	4.25
8-amino-2-naphthalenesulfonic acid	82ANSA	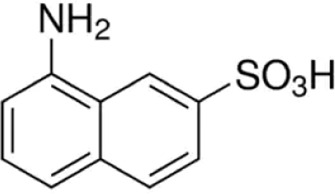	368; 465	0.96	NA

For each sample, 50 μg of dried IgG sample was resuspended and denatured by incubation with 30 μl SDS (1.33% wt/vol; Invitrogen, Carlsbad, CA, United States) at 65°C for 10 min. Subsequently, 10 μl of 4% Igepal-CA630 (Sigma-Aldrich, St Louis, MO, United States) and 1.2 u PNGase F (Promega, Madison, WI, United States) in 10 μl 5× PBS were added. The samples were incubated overnight at 37°C to allow release of N-glycans. The released N-glycans were labeled with the corresponding label. The labeling mixture was freshly prepared by dissolving the label (0.14 M) and 2-picoline borane (0.42 M) in a mixture of DMSO (Sigma-Aldrich) and glacial acetic acid (Merck, Darmstadt, Germany) (70:30, vol/vol). Labeling mixture (25 μl) was added to each N-glycan sample in the 96-well plate, which was then sealed using adhesive seal. Mixing was achieved by shaking for 10 min, followed by incubation at 65°C for 2 h. To each sample (75 μl), 700 μl of acetonitrile (ACN) (J. T. Baker, Phillipsburg, NJ, United States) was added. Free label and reducing agent were removed from the samples using HILIC solid-phase extraction (SPE). A GHP filter plate, 0.2 μm, (Pall Corporation, Ann Arbor, MI, United States) was used as the stationary phase. All wells were prewashed using 1 μl × 200 μl of ethanol/water (70:30, vol/vol) and 1 μl × 200 μl water, followed by equilibration using 1 μl × 200 μl of ACN/water (96:4, vol/vol). Solvent was removed by the application of a vacuum using a vacuum manifold (Millipore Corporation, Billerica, MA, United States). The samples were loaded into the wells, which were subsequently washed five times using 200 μl of ACN/water (96:4, vol/vol). Glycans were eluted with 2 μl × 90 μl of water and combined eluates were stored at −20°C until usage.

### HILIC-UPLC-FLR-MS analysis

Fluorescently labeled N-glycans were separated by HILIC on an Acquity H-class ultra-performance liquid chromatography (UPLC) instrument (Waters, Milford, MA, United States) consisting of a quaternary solvent manager, sample manager and a fluorescence detector (FLR) set with corresponding excitation and emission wavelengths for each glycan label (Table 1). The UPLC was coupled with a Synapt G2-Si ESI-QTOF-MS system (Waters) under the control of MassLynx v.4.1 software (Waters) in the first laboratory (Lab1), or with a Bruker Compact mass spectrometer with ionBooster source (Bruker Daltonics, Billerica, MA, United States) under the control of otofControl software (version 3.3, build 18; Bruker Daltonics) in the second laboratory (Lab 2). Labeled N-glycans were separated on a Waters bridged ethylene hybrid (BEH) Glycan chromatography column, 100 mm × 2.1 mm, 1.7 μm BEH particles, with 50 mmol/l ammonium formate, pH 4.4, as solvent A and ACN as solvent B. The separation method used a linear gradient of 75–62% acetonitrile (vol/vol) at flow rate of 0.4 ml/min in a 27 min analytical run. Samples were maintained at 10°C before injection and the separation temperature was 60°C. The injection volume was 40 μl.

MS conditions on the Synapt system (Lab1) were set as follows: negative ion mode, capillary voltage 2.5 kV, sampling cone voltage 30 V, source temperature 120°C, desolvation temperature 350°C, desolvation gas flow 600 l/h. Mass spectra were recorded from 500 to 3,000 m/z at a frequency of 1 Hz. MS conditions on the Bruker system (Lab2) were set as follows: negative ion mode, capillary voltage 2.5 kV, end plate offset 500 V, source temperature 150°C, vaporizer temperature 200°C, dry gas flow 4 L/min, nebulizer 5.5 bar. Mass spectra were recorded from 100 to 3,000 m/z at a frequency of 0.5 Hz.

In both laboratories MS parameters were optimized using 2-AB labeled glycan standard by direct-infusion ESI-MS in order to achieve maximum signal intensity. These parameters also proved to be optimal for the other tested labels.

### Mass spectrometry data quantification

MS data quantification was perfomed using MassLynx v.4.1 software (Waters) for data obtained in the first laboratory, while in the second laboratory Bruker Compass DataAnalysis 4.2 (Bruker Daltonics) was used. The 4 most intensive peaks were manually integrated in all samples: FA2 (biantennary N-glycan that contains terminal N-acetylglucosamine residues, and fucosylation on the core *N*-acetylglucosamine), FA2[6]G1 (biantennary N-glycan that contains one terminal galactose residue on the α1,6 arm, and fucosylation on the core N-acetylglucosamine), FA2G2 (biantennary N-glycan that contains terminal galactose residues and fucosylation on the core N-acetylglucosamine) and FA2G2S1 (biantennary N-glycan that contains one terminal sialic acid residue, and fucosylation on the core N-acetylglucosamine). Peaks were determined based on their MS/MS fragmentation spectra and previous results of automated integration approach (Pucić et al., 2011).

### Statistical analysis

Data was analyzed and visualized using the R programming language (version 3.5.2) and Microsoft Excel 2016 (Microsoft Corp., Redmond, WA, United States). Correlation of logP and RT with total area integrated was estimated using Kendall rank correlation coefficient. Considering multiple tests performed, false discovery rate (FDR) was controlled by the Benjamini–Hochberg method with *p*-value <0.05 considered as significant.

## Results

### HILIC-UPLC-FLR-ESI-MS analysis of differently labeled IgG N-glycans

**FIGURE 1:**
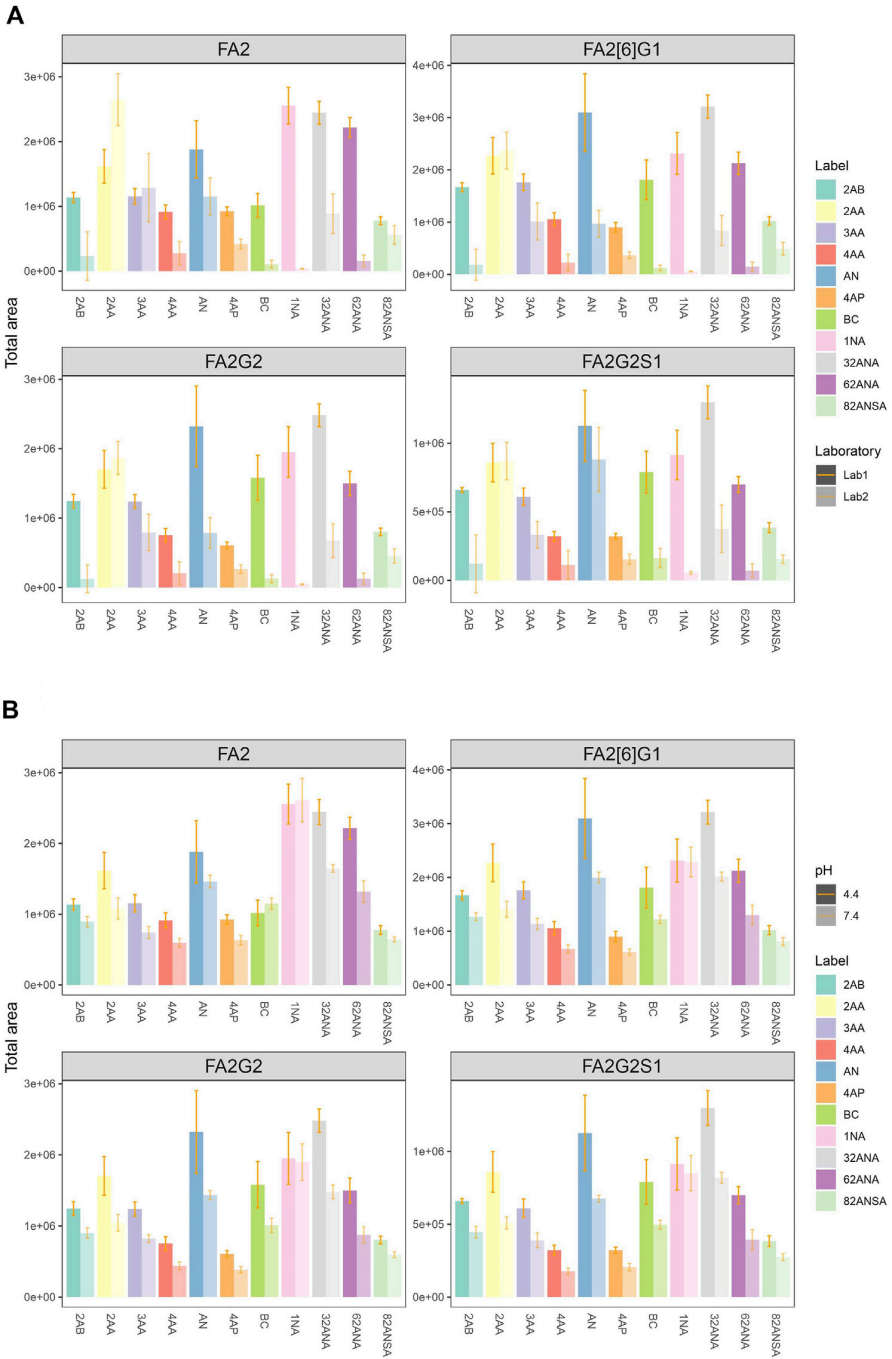
**(A)** Comparison of total area of all labels integrated for 4 most intensive peaks in two laboratories at pH 4.4. **(B)** Comparison of total area of all labels integrated for 4 most intensive peaks at pH 4.4 and 7.4 in the same laboratory (Lab1). Error bars represent the standard deviation of pentaplicates analysed.

**FIGURE 2:**
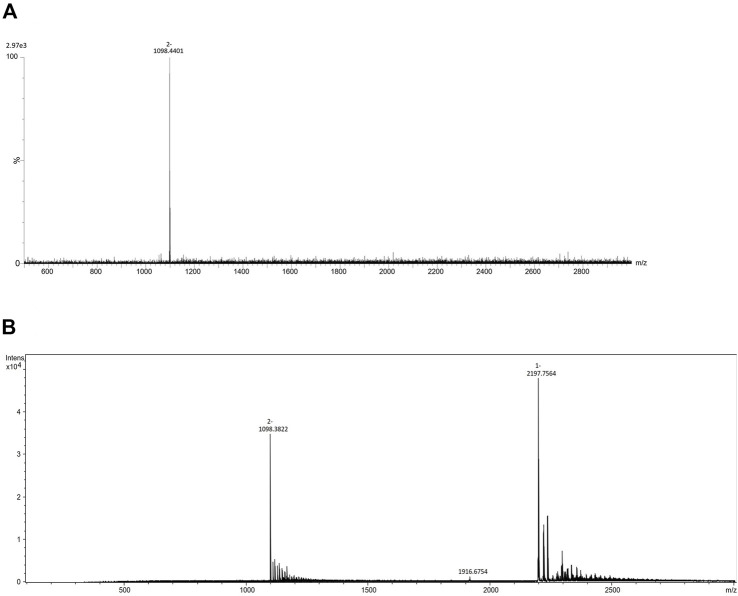
Representative sum spectra of 2AA labeled FA2G2S1 in **(A)** Lab1 and **(B)** Lab2.

Eleven fluorescent labels of different chemo-physical properties (Table 1) were chosen in order to study the influence of label hydrophobicity and presence of charge on N-glycan ionization in negative mode MS. N-glycans released from IgG sample were labeled with one of the eleven labels, purified with HILIC-SPE and analyzed with HILIC-UPLC-FLR-ESI-MS. To assure that negative MS mode really is superior to positive mode regarding the glycan structure elucidation on our instrument, we performed analysis of 2AB-labeled glycans in both modes, and found previously validated diagnostic ion for 6-arm mono-galactosylated FA2[6]G1 N-glycan structure (m/z 670.22) (Ashwood et al., 2018) only in negative MS mode (Supplementary Figure S1). Although we were primarily interested in improving the ionization efficiency and intensity of MS signals, we also wanted to make sure that these labels promote glycan detection in LC-FLR profiling. Therefore, both FLR and MS signals were obtained for each label. FLR excitation and emission wavelengths were experimentally determined for all of the labels individually, and all except 4-AP proved to be decent fluorescence tags (Supplementary Figure S2). LC separation of the IgG N-glycans was adequate and comparable among all labels. More hydrophobic labels showed shorter retention times, as was expected. To assess the label’s effect on MS signals 4 most intensive peaks were integrated in all samples: FA2, FA2[6]G1, FA2G2 and FA2G2S1, and their total area compared among the labels. In an effort to strengthen our conclusions on the ideal glycan label chemical characteristics for both LC and MS detection facilitation, analysis of the same samples was performed in two different laboratories and two different MS systems. The results are presented in Figure 1A. There were significant differences in labels’ effect on glycan ionization between laboratories: some of the labels with good signal intensities in one laboratory proved to have relatively low intensities in the other lab. For example, 1NA was one of the best performing labels in the first lab (Lab1), but was one of the worst performing in the other lab (Lab2). 2AA was by far the best label to use in the Lab2, while there were handful of labels with similar intensities in the Lab1. In Lab1 we found predominantly [M - 2H]^2−^ ions, while the Lab2 produced both [M - H]^−^and [M - 2H]^2−^ ions (Figure 2).

To further investigate performance of labels in negative mode MS, analysis was performed at both pH 4.4 and 7.4 in the same laboratory (Lab 1). The elevated pH resulted in significant signal drop among almost all labels and integrated peaks (Figure 1B). Only 1NA seemed to be not affected by the pH rise, retaining its beneficial qualities for ionization promotion.

### Labeling efficiency

To allow a fair comparison of the labels, we wanted to make sure that the differences in signal intensities are not coming from different labeling efficiency. Free, unlabeled glycans for most intensive IgG glycan peaks (FA2, FA2[6]G1, FA2G2, and FA2G2S1) were therefore detected by MS in all labeled samples. Differences in labeling efficiency were estimated based on the ratios of the total area of unlabeled and labeled glycans in each sample (Supplementary Table S1). For six of the labels, amount of free glycans was below LOQ (defined as S/N value of at least 10), suggesting maximum labeling efficiency. Four other labels (2AA, AN, 32ANA, 82ANSA) had neglectable amounts of unlabeled glycans, while only BC proved to have considerable amounts of free glycans. This indicates that ten out of eleven studied labels have fairly good and comparable labeling efficiency, with only BC standing out as somewhat poorer in effective tagging. Example comparison of labeled and unlabeled FA2 glycan for 2AA (good labeling efficiency) and BC (poor labeling efficiency) can be seen in Supplementary Figure S3.

### Effect of charge on ESI negative mode glycan ionization

**FIGURE 3:**
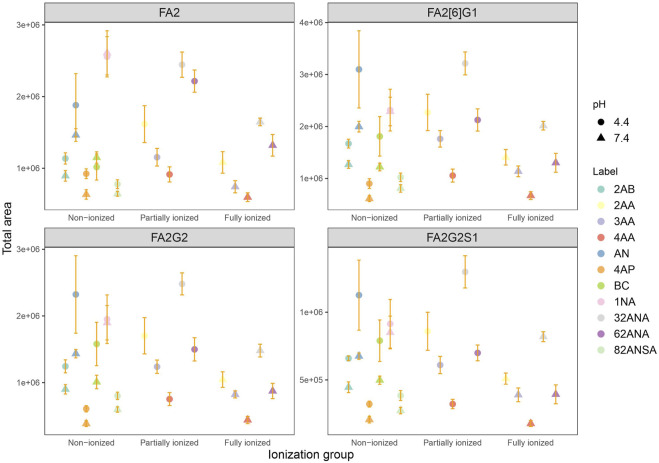
Comparison of total area of all labels grouped by their ionization for 4 most intensive peaks at pH 4.4 and 7.4 in the same laboratory. Error bars represent the standard deviation of pentaplicates analyzed.

Analysis was performed at both pH 4.4 and 7.4 for the purpose of studying the effect of label’s charge on ionization efficacy of glycans. With this dual pH approach, we could not only compare neutral to ionized labels, but also study intensity change of an individual label as its dissociation progresses. For the sake of simplicity of interpretation, labels were divided into three groups based on their dissociation on a given pH: non-ionized (neutral), partially ionized (25–60% dissociated) and fully ionized (100% dissociated). Six of the labels chosen are neutral at pH 4.4, while five are partially ionized (25–60%). At pH 7.4, five labels are fully ionized while the remaining six are completely neutral (Table 2). Figure 3 shows no apparent trend among the different labels grouped based on their dissociation regarding total area integrated. Furthermore, labels which became fully ionized at pH 7.4 suffered from signal drop and similar effect was seen for neutral labels, suggesting that presence of charge on the label has no beneficial effect on ionization efficacy of the derived glycan. On that note, signal differences of structurally similar labels such as 2AA-3AA-4AA or 32ANA-62ANA could be explained by subtle differences between their pKa values. It can be seen that more dissociated labels (that is, labels with smaller pKa) gave lower signal intensities than more neutral labels.

**TABLE 2:** Percentage of label ionized at pH 4.4 and 7.4. Percentage of ionization was calculated based on the pKa values using Henderson-Hasselbalch equation.

Label	pKa	Dissociation at pH 4.4 (%)	Dissociation at pH 7.4 (%)
2AB	NA	NA	NA
2AA	4.89	24.5	100
3AA	4.81	28.0	100
4AA	4.77	29.9	100
AN	NA	NA	NA
4AP	10.4	0	0.001
BC	NA	NA	NA
1NA	NA	NA	NA
32ANA	4.76	30.4	100
62ANA	4.25	58.6	100
82ANSA	NA	NA	NA

### Effect of hydrophobicity on ESI negative mode glycan ionization

**FIGURE 4:**
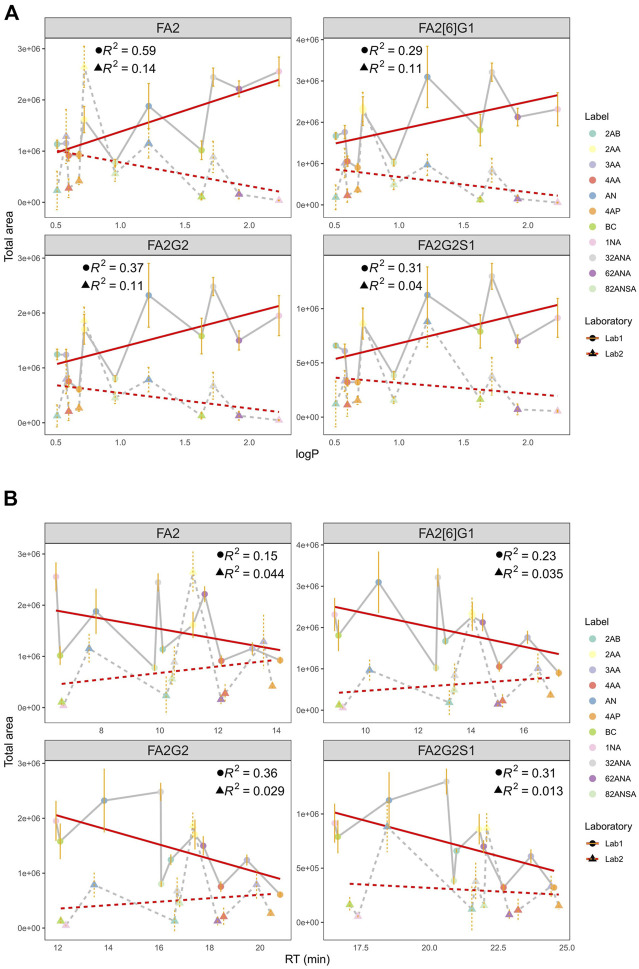
**(A)** Correlation of total area and logP at pH 4.4 in two laboratories for all labels integrated for 4 most intensive peaks. **(B)** Correlation of total area and RT at pH 4.4 in two laboratories for all labels integrated for 4 most intensive peaks. Error bars represent the standard deviation of pentaplicates analysed.

As a measure of labels’ hydrophobicity, partition coefficient was chosen and is presented in Table 1 as logP. Interestingly, labels affected the glycan ionization quite differently on different MS systems in two different labs (Figure 4A). While in Lab1 there is a noticeable trend of more hydrophobic labels having bigger signal intensities, no such trend is present in the other laboratory. Labels with higher logP generally had beneficial effect on glycan ionization in negative mode MS in Lab1, but that was not the case in Lab2. For example, 1NA, as the most hydrophobic label used, had among the best signal intensities in Lab1, but was one of the poorest performing labels in Lab2. The correlation of logP and total area integrated proved to be statistically significant for all the measured glycans in Lab1 (Supplementary Table S2), with *R* ^2^ ranging from 0.29 for FA2[6]G1 (*p* = 6.6 × 10^−4^) to 0.59 for FA2 (*p* = 7.0 × 10^−5^). Same effects in Lab1 could be seen for both pH 4.4 and 7.4 (Supplementary Table S2). On the other hand, Lab2 seems to have the opposite trend, yet smaller effects, which did not reach statistical significance.

Additionally, hydrophobicity trends were re-evaluated using retention time (RT) as a measure of labels’ hydrophobicity (Figure 4B). More hydrophobic labels have smaller retention times on the HILIC column, and opposite is the case for more hydrophilic ones. Similar tendency can again be seen: more hydrophobic labels tend to have higher signal intensities in Lab1, while that is not the case in Lab2. Negative correlation of RT and total area proved to be statistically significant in Lab1 (Supplementary Table S3), with *R* ^2^ ranging from 0.15 for FA2 (*p* = 3.3 × 10^−2^) to 0.36 for FA2G2 (*p* = 5.3 × 10^−5^). Again, this tendency was seen for both pH 4.4 and 7.4 (data not shown).

## Discussion

Characterization and quantitation strategies for N-glycan analysis often require a derivatization step in order to prevail different problems accompanying such challenging analytes. Namely, as glycans themselves lack chromophores or fluorophores, their optical detection is practically impossible. In addition, given rather poor ionization properties of native glycans, their mass-spectrometric detection is also rarely satisfactory. Introduction of a labeling tag is a common and pretty successful way of dealing with such challenges. Labels are most commonly attached to glycans *via* process called reductive amination through Schiff base formation, and various tags have been used for this purpose (Ruhaak et al., 2010; Zhou et al., 2017). The most widely applied ones are 2-aminobenzamide (2-AB) and 2-aminobenzoic acid (2-AA), the latter being considered as somewhat of a standard for negative mode MS (Harvey, 2020; Lattová et al., 2005).

A prosperous tag would enhance both LC and MS detection, as well as have beneficial effect on chromatographic separation. In order to find the optimal chemical structure of a glycan label, we tested eleven different fluorescent labels and assessed their impact on negative mode MS ionization. As mass spectrometric analysis of glycosylation is generally done in positive mode MS, there have been a couple of studies inspecting effects of different labels on ESI-MS positive mode ionization (Harvey, 2000; Keser et al., 2018: Lattová et al., 2005; Walker et al., 2010). Although some of the studies were also done in negative mode, they were done on only a few labels and without a focus on their hydrophobicity (Lattová et al., 2005; Lang et al., 2021). Our selection of 11 labels included a range of hydrophobicity of the tags (logP 0.51—2.23) and different ionization groups. Herein we present a side by side comparison of total area integrated for 4 most intensive glycan peaks for all the labels, under two pH and in two laboratories.

In order to determine the effect of mobile phase pH on negative mode ESI-MS signals, analysis was performed at pH 4.4 and 7.4. Studies done in positive mode MS showed that high mobile phase pH could be beneficial for analyzing small pharmaceutical molecules (Kafeenah et al., 2019), but also presented that some compounds are not pH-dependent (Liigand et al., 2017). These studies concluded that effect of mobile phase pH on molecules’ ionization efficiency cannot be predicted solely on the degree of its ionization or pKa (Liigand et al., 2017). Although in theory pH value 2 units above pKa value of the acidic analyte sounds ideal for negative mode as it would become completely deprotonated thus producing negative ions, we observed completely opposite trend. Although the extent of the ionization efficiency change with pH was not the same for all the labels, there was a significant signal decrease among almost all labels and integrated peaks after switching to pH 7.4 instead of pH 4.4. 1NA proved to be pH-independent, promoting the ionization efficiently even on the elevated pH. Interestingly, partially and fully ionized labels at pH 7.4 suffered from a signal drop comparable to those of non-ionized tags. Progression of dissociation on the elevated pH therefore seemed to have no additional detrimental effect on ionization efficacy, suggesting that the presence of charge is not crucial for a successful label. Nevertheless, an obvious trend of signal drop among structurally similar labels such as 2AA-3AA-4AA and 32ANA-62ANA, possibly explained by their different pKa values and resulting dissociation differences, indicates that presence of negative charge on the label actually has unfavourable effect on glycan ionization in negative mode MS.

Except for presence of charge, we were also interested in hydrophobicity of the studied labels. Hydrophobic tagging in ESI proteomics has long been utilized in order to substantially increase electrospray response, therefore decreasing detection limits (Shuford and Muddiman, 2011). Similar approaches have been incorporated in glycomics, with studies showing that inclusion of hydrophobic surface area to the glycan structure increases its ion abundance (Walker et al., 2010, 2011). As those studies were done in positive mode MS, we wanted to test this effect in negative mode ESI-MS in two laboratories. In Lab1, we detected a clear trend towards better ionization efficiency with more hydrophobic labels, as expected. Best performing labels proved to be AN, 1NA, 32ANA and 62ANA. We anticipated to observe similar effects in Lab2, but surprisingly the trend was not present. Even the best performing labels differed: 2AA was without a doubt a label to opt for in Lab2, although being one of the most hydrophilic ones tested. This data speaks in favour of a huge effect of subtle changes in mass spectrometry conditions which makes interlaboratory comparisons pretty challenging, as reported before (Clark et al., 2021). One MS source parameters can favour ionization of one label, while the other source can cause ionization enhancement of some other label. As previously reported, MS parameters and intrinsic configuration of the spectrometer could have an effect on the final ESI response (Kiontke et al., 2016).

Comparing the ionization behaviours of different derivatives in two laboratories, we observed that in Lab1 the most abundant ions are doubly charged [M - 2H]^2-^, while the Lab2 produced both [M - H]^-^ and [M - 2H]^2-^. This ionization pattern proved consistent for all the labels (Figure 2). These changes in charge states of the most abundant ions as well as differences in adduct formations are presumably a consequence of different ionization sources between the laboratories. Moreover, as we have obtained almost opposite trends between laboratories, it seems that ionization source has bigger effect on labeled-glycan ionization in negative MS mode than the labels themselves. Fine-tuning of MS parameter settings, such as voltage and temperature, has already been reported as an approach for 3-fold increase of N-glycan ion abundances (Hecht et al., 2015). Although chosen labels are not so different in structure, obviously there is a need to optimize MS parameters for every label in order to achieve maximum increase in ion abundances. Therefore, search for an ideal label should be done in all laboratories individually, tuning the spectrometer settings specifically for the MS system used. Although finding the ideal label for glycan ionization promotion may be impossible, finding a suitable label for the job in a given laboratory could be done.

## Data Availability

The raw data supporting the conclusion of this article will be made available by the authors, without undue reservation.

## Supplementary material

SUPPLEMENTARY FIGURE S1 Mass spectra of mono-galactosylated N-glycan with previously validated diagnostic product ion for 6-arm composition highlighted: **(A)** FA2[6]G1 in negative MS mode, **(B)** FA2[3]G1 in negative MS mode, **(C)** FA2[6]G1 in positive MS mode, **(D)** FA2[3]G1 in positive MS mode. In negative MS mode, the presence of the diagnostic ion on the glycan with 6-arm (FA2[6]G1) has more than one order of magnitude higher intensity compared to the 3-arm isomer (FA2[3]G1).

SUPPLEMENTARY FIGURE S2 Fluorescence signal to noise ratio of all the labels.

SUPPLEMENTARY FIGURE S3 Extracted ion chromatogram of labeled and unlabeled FA2 glycan for **(A)** 2AA and **(B)** BC.

## Abbreviations

FA2, biantennary N-glycan that contains terminal N-acetylglucosamine residues, and fucosylation on the core N-acetylglucosamine; FA2[6]G1, biantennary N-glycan that contains one terminal galactose residue on the α1,6 arm, and fucosylation on the core N-acetylglucosamine; abrFA2G2, biantennary N-glycan that contains terminal galactose residues and fucosylation on the core N-acetylglucosamine; FA2G2S1, biantennary N-glycan that contains one terminal sialic acid residue, and fucosylation on the core N-acetylglucosamine; FLR detector, fluorescence detector; HILIC, hydrophilic interaction chromatography.
