# Supplementary figures and images for: In a pursuit of optimal glycan fluorescent label for negative MS mode for high-throughput N-glycan analysis
Source: Front Chem. 2022 Oct 3;10:999770. doi: 10.3389/fchem.2022.999770 (PMC9574008; doi:10.3389/fchem.2022.999770)

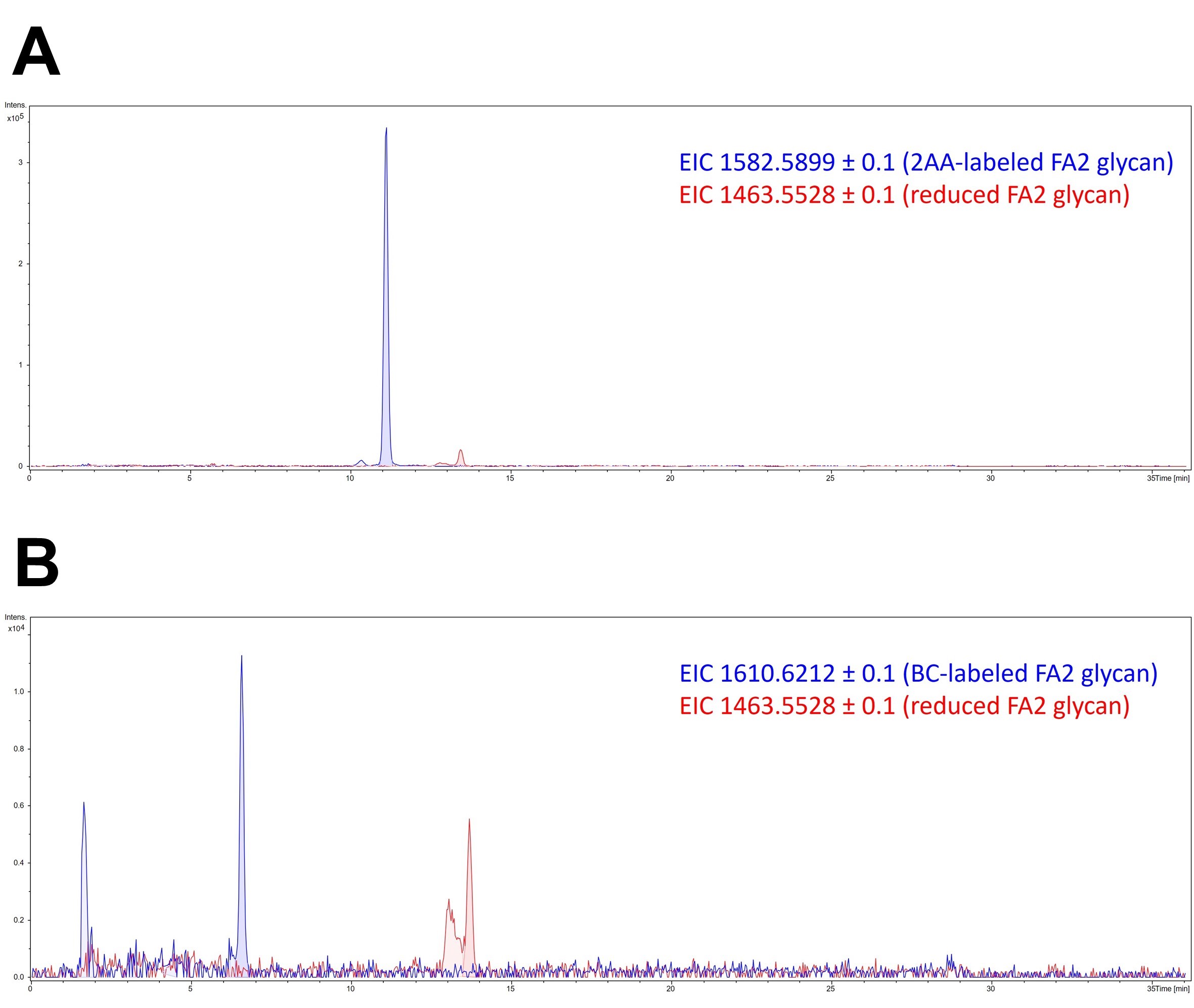

Supplement: Supplementary file 2 [file Image3.JPEG]

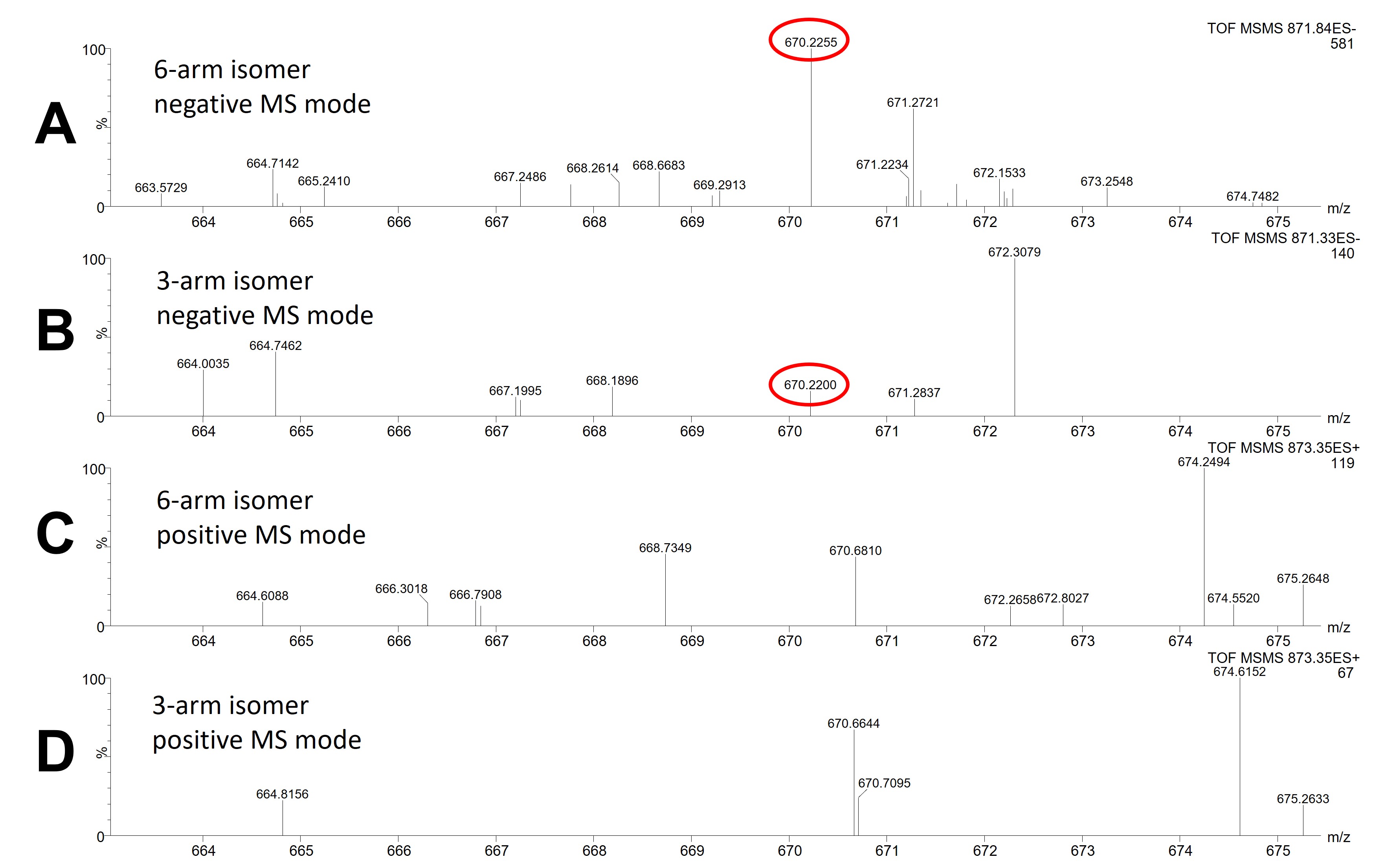

Supplement: Supplementary file 4 [file Image1.JPEG]

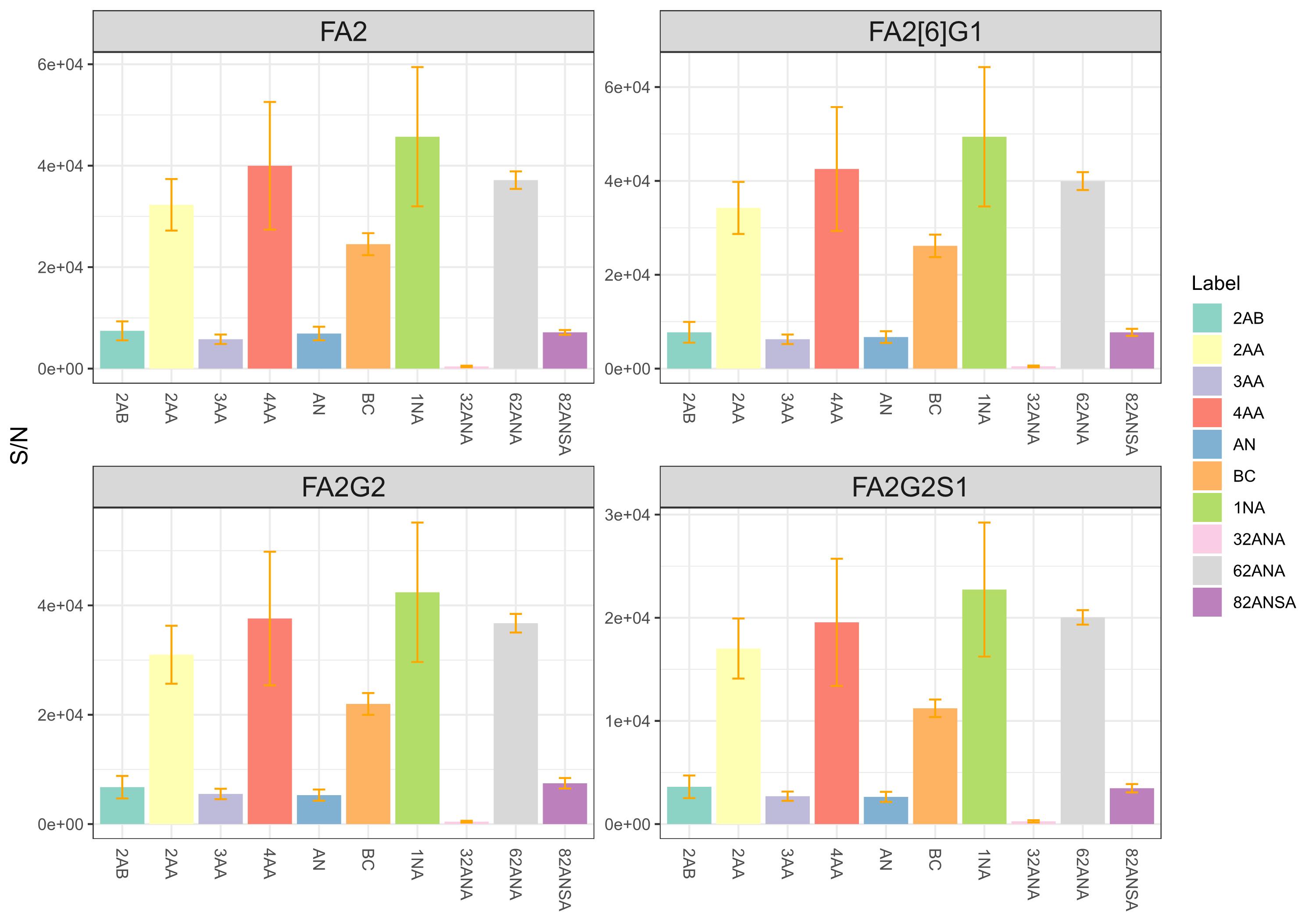

Supplement: Supplementary file 5 [file Image2.JPEG]
